# Editorial: Advance in B-cell therapies for the treatment of rheumatic and musculoskeletal diseases

**DOI:** 10.3389/fmed.2022.1020859

**Published:** 2022-09-21

**Authors:** Md Yuzaiful Md Yusof, Stefano Alivernini, Katerina Chatzidionysiou

**Affiliations:** ^1^Leeds Institute of Rheumatic and Musculoskeletal Medicine, University of Leeds, Leeds, United Kingdom; ^2^National Institute for Health and Care Research (NIHR) Leeds Biomedical Research Centre, Leeds Teaching Hospitals National Health Service (NHS) Trust, Leeds, United Kingdom; ^3^Immunology Core Facility, Gemelli Science Technological Park, GSTeP, Fondazione Policlinico Universitario A. Gemelli Istituto di Ricovero e Cura a Carattere Scientifico (IRCCS), Rome, Italy; ^4^Division of Rheumatology, Fondazione Policlinico Universitario A. Gemelli Istituto di Ricovero e Cura a Carattere Scientifico (IRCCS)—Università Cattolica del Sacro Cuore, Rome, Italy; ^5^Rheumatology Unit, Department of Medicine, Karolinska Institute, Stockholm, Sweden

**Keywords:** B lymphocyte, biological DMARDs, rheumatoid arthritis, Sjögren's syndrome, systemic lupus erythematosus

In this Research Topic of Frontiers in Medicine, our aim was to highlight advances in B-cell therapies in various rheumatic and musculoskeletal diseases (RMD) to further refine their use in clinical trial and routine practice. The two most evaluated strategies for B-cell blockade for the treatment of rheumatic and musculoskeletal diseases (RMD) over the last three decades are B-cell depletion and inhibition of B-cell survival factors i.e., B-cell-activating factor (BAFF) and/or A proliferation-inducing ligand (APRIL). Rituximab is a chimeric anti-CD20 monoclonal antibody (mAb). The depth of B-cell depletion and clinical responses may vary, implying potential pathogenic or pharmacodynamic differences between subgroups of patients. Recent data have supported the efficacy of reduced rituximab dose and the different retreatment strategies in its licensed indications; rheumatoid arthritis (RA), granulomatosis with polyangiitis/microscopic polyangiitis and pemphigus vulgaris, although long-term data are still needed to establish the optimal approach (1–5). Despite failure of rituximab in meeting its primary endpoints when investigated in systemic lupus erythematosus (SLE) (6, 7) and primary Sjogren's syndrome (pSS) (8, 9), it may still be used in refractory cases based on clinical effectiveness from case series (10–13). Belimumab, a BAFF-inhibitor, is licensed for patients aged ≥5 years with active, autoantibody-positive SLE who are receiving standard therapy and those aged ≥18 with active lupus nephritis who are receiving standard therapy. Its action on both B-cells and non-B-cells may have contributed to the success of belimumab trials. There remains an unmet need for mechanistic and clinical studies concerning stratification of patients who would respond best to both rituximab and belimumab to aid personalized therapy.

In RA, the optimal retreatment paradigm for rituximab has not been fully determined. Three strategies are commonly used (3). (i) Fixed retreatment e.g., 2 × 1,000 gm infusions administered every 6 months. Nevertheless, regular retreatment may risk overtreatment in some patients and increases the risk of infections associated with secondary hypogammaglobulinaemia (14). (ii) Retreatment can also be employed based on treat-to-target approach, in line with the European League Against Rheumatism (EULAR) recommendations for RA management whereby target of treatment is remission [DAS28 <2.6, Simple Disease Activity Index (SDAI) <3.3 or Clinical Disease Activity Index (CDAI) <2.8 or at least low disease activity (LDA)] (15). (iii) Retreatment-on-clinical relapse or “on demand.” Inherent to this is a degree of instability, with potential clinical implications, such as more short-term corticosteroid use that can be potentially detrimental to long-term outcomes. This could be improved by identifying clinical and biomarkers of imminent relapse. Kim et al. used data from the Korean Rheumatology Biologics registry (KOBIO) and patients who were treated at the Ajou University Hospital, Suwon South Korea. Eighty-two patients were enrolled and those who responded were treated on demand. The mean time-to-retreatment was 16 months. In multivariable analysis, factors associated with longer time-to-retreatment were concomitant use of 2 or more csDMARD and concomitant use of corticosteroid (16). The latter should be interpreted with caution since there was no consistent association with time-to-retreatment when concomitant daily oral prednisolone dose was evaluated. At 5 years, the rituximab retention rate was 72% which was a good outcome from therapeutic perspective. Since some patients appear to be refractory to B-cell depleting therapy in RA, another therapeutic option is through the Janus kinase (JAK) inhibition. Moura and Fonseca discussed in a narrative review article that currently available JAK inhibitors (tofacitinib, baricitinib, upadacitinib, peficitinib, filgotinib, and decernotinib) can affect B-cell activation, proliferation and differentiation and could be beneficial in the pre-clinical or early phase of RA (17).

In pSS, Pavlych et al. conducted a retrospective observational cohort study to compare the effectiveness of rituximab originator (MabTherara^®^) and rituximab biosimilar (Truxima^®^) in patients with a disease duration of <5 years and a systemic moderate–high activity [as defined by The EULAR Sjögren's syndrome (SS) disease activity index (ESSDAI) ≥5 points]. Nine and eight patients were treated with the originator and the biosmilar, respectively. At 48 weeks, the mean ESSDAI score was significantly reduced compared to pre-rituximab score in all patients and there was no difference in the change in ESSDAI score from baseline between both treatment arms. Disappointingly, there was no improvement observed in the change in a patient-reported outcome, the EULAR Sjogren's Syndrome Patient Reported Index (ESSPRI) in all patients at weeks 24 and 48 from baseline and between the treatment groups (18).

In SLE, Wise and Stohl wrote a narrative review article and discussed the outcome disparities in randomized controlled trials (RCTs) between rituximab and belimumab. Failure of rituximab in meeting its primary endpoint could be attributed to its poor trial design and to a degree its biological effect i.e., plasma cells do not express CD20 and thus, are insensitive to rituximab. In contrast, in addition to a better trial design including the use of a new composite primary endpoint, the SLE Responder Index (SRI-4) and adequate sample size, plasma cells express B-cell maturation antigen (BCMA) and TNF receptor superfamily member 13b (TACI) of which both are inhibited by belimumab, thus abate ongoing pathogenic autoantibody production by plasma cells (19). These factors could influence the success of belimumab in five RCTs. *Post-hoc* analysis of belimumab RCTs and real-world observational studies identified characteristics of patients would most likely to respond to belimumab including those with high disease activity [the SLE Disease Activity Index 2000 (SLEDAI-2K) ≥10], anti-dsDNA positivity, low complement levels, polyarthritis, non-smoking status, and lack of significant end organ damage (20–22). Plüß et al. reported case series of seven patients who were treated with belimumab for non-approved SLE features (renal = 6 and neuropsychiatric = 1). Following therapy with belimumab, proteinuria was markedly improved in all patients and one patient with dysarthria and ataxia improved (23). Belimumab plus standard therapy has since approved for active lupus nephritis following a positive outcome in a phase III RCT (24). Nevertheless, it is important to note that the effect size of belimumab over its comparator in RCTs overall was rather modest (ranging from 9.7 to 14%), as well as an RCT in patients of black African ancestry failed to meet its primary endpoint at 52 weeks (25). Another subgroup of patient who may not respond well to belimumab is those who develop secondary non-depletion non response (2NDNR) to rituximab which is associated with anti-rituximab antibodies (26). Hassan et al. conducted an observational cohort study and compared the effectiveness of switching those with 2NDNR to rituximab to either belimumab (*N* = 8) or an alternative humanized anti-CD20 agent (*N* = 6; ocrelizumab = 3, ofatumumab = 2, obinutuzumab = 1). All patients in the latter group achieved SRI-4 response while only 1/8 patient in the former group met SRI-4 response. Moreover, 2/8 patients in the former developed lupus nephritis including one *de novo* Class II and V nephritis (27). This study suggests that patients who developed 2NDNR to rituximab should be switched within the same biologic class i.e., humanized or type 2 anti-CD20 mAbs.

In a narrative review article, Parodis et al. discussed other promising strategies to improve B-cell blockade in SLE including plasma cell inhibition using proteasome inhibitor such as bortezomib, the next generation anti-CD20 mAbs including obinutuzumab, targeting B-cell intracellular signaling through inhibition of Bruton's Tyrosine Kinase (BTK) and the use of chimeric auto-antigen receptor (CAAR) T-cells that have been genetically engineered to kill human autoreactive B-cells (28). They also discussed since BAFF level rose following treatment with rituximab, combining therapy of rituximab and belimumab would be a logical approach. This was supported by the results from BEAT-LUPUS RCT where add on add-on belimumab was superior over rituximab alone in prolonging the time-to-severe SLE flare and in reducing anti-dsDNA antibody titres (29). Another alternative is sequential therapy for which Petricca et al. reported a case report of a patient with severe and refractory lupus nephritis and bullous pemphigus who responded to treatment with rituximab, followed by belimumab (30).

Finally from B-cell biomarker perspective, You et al. compared peripheral blood mononuclear cells of 57 SLE patients and 50 healthy controls using flow cytometry. They found that double negative B-cells (DN Bcells; CD19+CD27–lgD–) were associated with lupus nephritis and positively correlated with proteinuria level. The proportion of lupus nephritis patients who achieved remission following a therapy was higher in SLE patients with low DN Bcells than in patients with high pre-treatment DN Bcells rates; 83 and 25% respectively, thus could be a promising prognostic biomarker in lupus nephritis (31).

We thank the contributing authors for shedding light on the advance in B-cell therapies in our Research Topic, with the ultimate goal of improving the care of patients with RMD. Future research agenda will build on this progress and should focus on better biomarkers that may allow prediction of active disease, prognosis and/or response to therapy through the application of new technologies and clinical efficacy of novel B-cell-targeted therapies with stratification of therapy to disease manifestation and their long-term safety particularly in terms of the risk of severe infection and major cardiovascular events.

## Author contributions

MM wrote the first draft of the Editorial, which was then reviewed by SA and KC who revised it critically for important intellectual content and final approval of the version published. All authors have agreed to be accountable for all aspects of the work in ensuring that questions related to the accuracy or integrity of any part of the work are appropriately investigated and resolved.

## Conflict of interest

MM has received consultancy fees from Aurinia Pharmaceuticals and UCB. KC received consultancy fees from Eli Lilly, AbbVie, and Pfizer. The remaining author declares that the research was conducted in the absence of any commercial or financial relationships that could be construed as a potential conflict of interest.

## Publisher's note

All claims expressed in this article are solely those of the authors and do not necessarily represent those of their affiliated organizations, or those of the publisher, the editors and the reviewers. Any product that may be evaluated in this article, or claim that may be made by its manufacturer, is not guaranteed or endorsed by the publisher.

## Author disclaimer

The views expressed are those of the author(s) and not necessarily those of the NIHR or the Department of Health and Social Care.
